# Late Miocene megalake regressions in Eurasia

**DOI:** 10.1038/s41598-021-91001-z

**Published:** 2021-06-01

**Authors:** Dan Valentin Palcu, Irina Stanislavovna Patina, Ionuț Șandric, Sergei Lazarev, Iuliana Vasiliev, Marius Stoica, Wout Krijgsman

**Affiliations:** 1 Paleomagnetic Laboratory Fort Hoofddijk, Department of Earth Sciences, Utrecht University, Utrecht, The Netherlands; 2 Oceanographic Institute, University of Sao Paulo, Sao Paulo, Brazil; 3 Geological Institute of the Russian Academy of Science, Moscow, Russia; 4 Faculty of Geography, University of Bucharest, Bucharest, Romania; 5 Senckenberg Biodiversity and Climate Research Centre (SBiK-F), Frankfurt am Main, Germany; 6 Department of Geology, University of Bucharest, Bucharest, Romania

**Keywords:** Palaeomagnetism, Geology

## Abstract

The largest megalake in the geological record formed in Eurasia during the late Miocene, when the epicontinental Paratethys Sea became tectonically-trapped and disconnected from the global ocean. The megalake was characterized by several episodes of hydrological instability and partial desiccation, but the chronology, magnitude and impacts of these paleoenvironmental crises are poorly known. Our integrated stratigraphic study shows that the main desiccation episodes occurred between 9.75 and 7.65 million years ago. We identify four major regressions that correlate with aridification events, vegetation changes and faunal turnovers in large parts of Europe. Our paleogeographic reconstructions reveal that the Paratethys was profoundly transformed during regression episodes, losing ~ 1/3 of the water volume and ~ 70% of its surface during the most extreme events. The remaining water was stored in a central salt-lake and peripheral desalinated basins while vast regions (up to 1.75 million km^2^) became emergent land, suitable for development of forest-steppe landscapes. The partial megalake desiccations match with climate, food-web and landscape changes throughout Eurasia, although the exact triggers and mechanisms remain to be resolved.

## Introduction

### A tectonically trapped sea turns into a megalake

**Figure 1:**
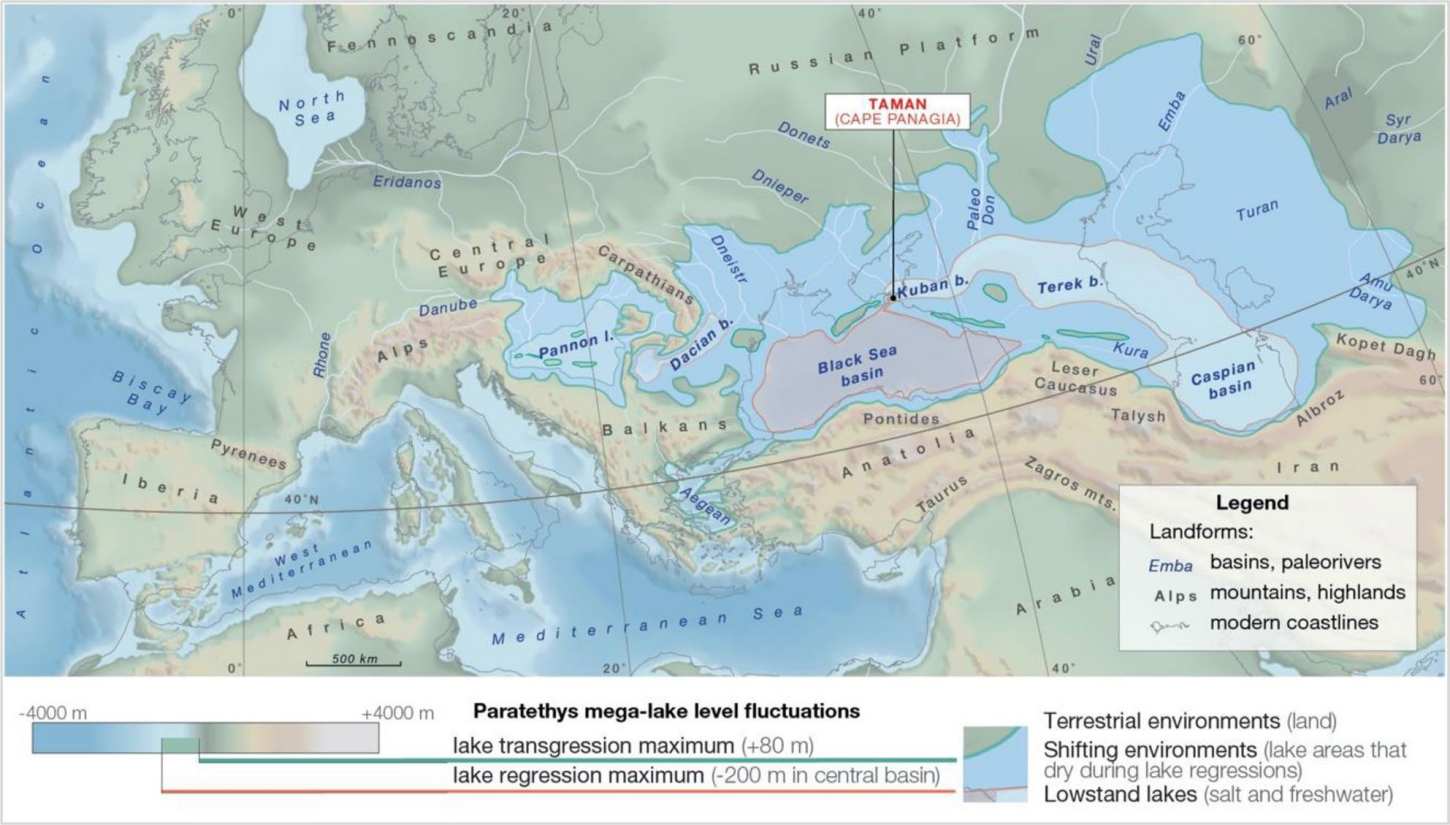
Paleogeographic reconstruction of late Miocene Paratethys fluctuations. During regressive phases, the megalake lost most of its surface. The remaining water was split between a central salt-lake in the Black Sea basin (marked in red) and peripheral basins that periodically refilled and became fresher (light blue) (Map generated in ArcGIS Pro https://www.esri.com/en-us/arcgis/products/arcgis-pro/overview, v. 2.1, see “Methods”).

At the beginning of the late Miocene (11.6 Ma) the European continent was very different from today (Fig. 1). In the west it was separated from Africa by an archipelago^1,2^. To the south the Anatolian—Balkan landmass was splitting apart, giving way to lakes that would become the modern Aegean Sea^3,4^, while the landmasses of modern Italy were scattered in a cluster of islands in the central Mediterranean Sea^5,6^. However, the most striking feature was the Paratethys—a water body the size of the Mediterranean that stretched between the Eastern Alps and modern Kazakhstan^6,7^.

The Paratethys was initially an epicontinental sea that formed at the beginning of the Oligocene (~ 34 Ma) from the remnants of the northern Tethys Ocean^8^. The sea was characterized by progressive fragmentation, gateway restrictions and sediment filling, mostly due to tectonics^9^. Progressive uplift of the central European mountain ranges gradually isolated the Paratethys from the global ocean. At the onset of the late Miocene, the ancient sea transformed into a megalake characterized by anomalohaline salinities (generally ranging between 12–14‰)^10^ and developed a unique endemic fauna (e.g., mollusks and ostracods). The landlocked Paratethys underwent extreme hydrological crises and partial desiccation episodes^7^. This had devastating effects on the aquatic fauna as the diversity of the endemic ecosystem was greatly reduced and multiple groups, such as foraminifera and nannoplankton, disappeared almost entirely^11,12^. The wider impacts and implications of these hydrological crises, in particular beyond the Paratethys area, are still poorly understood.

### Chronology of late Miocene megalake regressions in Eurasia

Upper Miocene Paratethys sedimentary successions are exposed extensively in Eurasia but are often incomplete or interrupted by continental deposits (Fig. S1). In the western Black Sea region, carbonate platform deposits crop out and are truncated by repeated erosion and karstification episodes^13^, while in the northwestern region riverine-lacustrine successions of an extensive deltaic system developed^14^. Well-preserved aquatic records have been reported in peripheral basins of the southwest Caspian^7^ and south Carpathians^15^, but limited biostratigraphic information prevents development of integrated stratigraphies in these regions. More suitable fossiliferous sections are available on the eastern Crimean and western Taman peninsulas^16^. The classical Neogene sections of Taman were first described at the end of the nineteenth century and beginning of the twentieth century and have been used as reference sections for development of the Eastern Paratethys regional stratigraphic chart^17^. Attempts to retrieve cores of the late Miocene record from the deeper central Black Sea basin during Deep-Sea Drilling Project (DSDP) Leg 42b had limited success, leaving the Neogene sections on the Black Sea coast of Taman (Fig. 1) as the only viable option for developing a chronology of late Miocene megalake fluctuations.

Taman Peninsula, Russia (Figs. 1, S2), contains several key stratigraphic sections^18–20^, including Cape Panagia (45°15′57.81″N/26°25′31.91″E), the only place known to host a continuous sedimentary record of the late Miocene Paratethys hydrological crises^7,15,16^. The sedimentary succession of Cape Panagia (Fig. S3) belongs to the Kuban basin^20^, adjacent to the Black Sea basin, the central and deepest part of the Paratethys. The section has been subject to extensive lithological and paleontological investigations, synthesized in a recent monograph^16^ that we use as a litho-biostratigraphic reference. Our sedimentologic analyses (see “Methods” and Fig. S4), correlated with biostratigraphic data^16^, indicate that the Panagia section is characterized by deep-water sediments, interrupted by four major water-level regressions identified throughout the Paratethys^7^.

The first regression interval is ~ 50 m thick and contains organogenic carbonate buildups (bryozoan-algal and microbial-algal mats)^21^, interbedded with finely laminated dolomitized limestones^22^ and clays (Figs. S2–S5, Table S1). Biostratigraphic correlations indicate that this interval corresponds to a significant base-level fall, freshening and shrinking of the basin^7,16^. According to estimations of Paratethys water level fluctuations and shore migrations^7^, this regression involved a ~ 230 m drop (from + 80 to − 150 m). It corresponds to the collapse of saltwater Paratethys fauna at the regional Bessarabian-Khersonian substage boundary^16^. Multiple faunal groups disappeared, and the endemic fauna was greatly reduced, indicating a significant biological crisis for aquatic life. The second regression phase contains organogenic carbonate buildups (bryozoan-algal mats^21^), interbedded with finely laminated limestones and clays, indicating paleobathymetric reductions alternating with incomplete recovery phases. This regression is not clearly described in the literature, likely because it occurred shortly after the previous episode. Based on its lithological expression we tentatively estimate a lowstand similar to the first event. Carbonate levels, similar to the previous levels but less developed are correlated to a third regression documented in Paratethys, when water levels dropped by ~ 100 m (from + 50 to − 50 m)^7^. The fourth and final regression is the most severe and is known as the Great Khersonian Drying^15^ (event kd, in Fig. 3, Fig. S4). It corresponds to a water level drop of more than 250 m (from + 50 to > − 200 m) at the end of the Khersonian regional stage, when Panagia environments became terrestrial for a brief time. The Maeotian transgression^7^ (event mt, in Fig. 3, Fig. S4), widely documented throughout Eurasia, marks the end of this dry period and progressively refilled the basin^15^.

**Figure 2:**
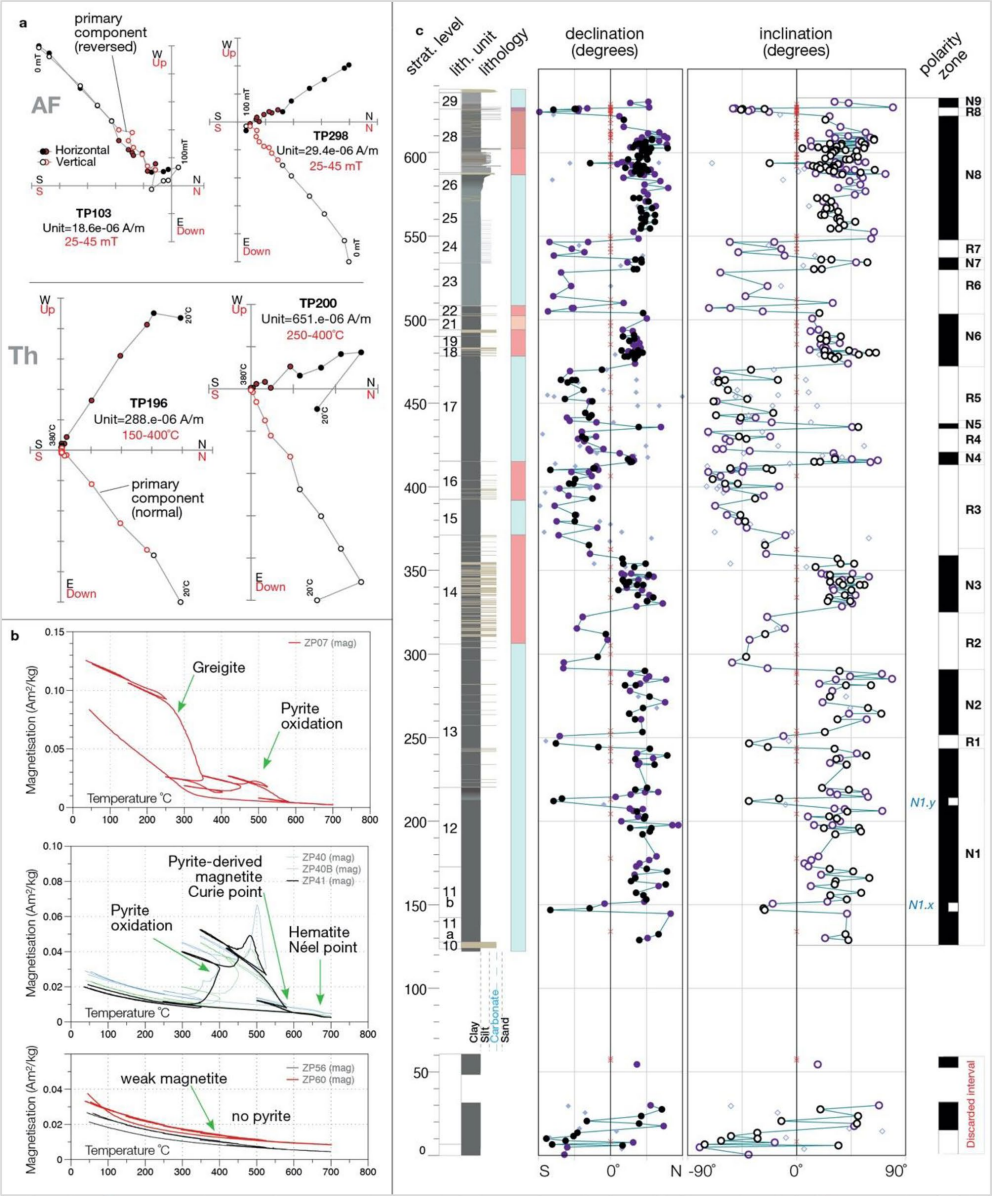
Paleomagnetic results from Cape Panagia. (**a**) Examples of demagnetization plots with normal and reversed polarities (*AF* alternating field, *Th* thermal demagnetization) with intervals used for interpretation highlighted in red; (**b**) Thermal behaviour of samples from Cape Panagia, obtained from Curie balance experiments with multiple cooling and heating steps; (**c**) Magnetic directions plotted in stratigraphic order and local magnetic polarity pattern. Log and lithological unit numbers are edited, from Popov et al.^16^; the coloured bar indicates Paratethys high-stands (light blue) and low-stands (red).

**Figure 3:**
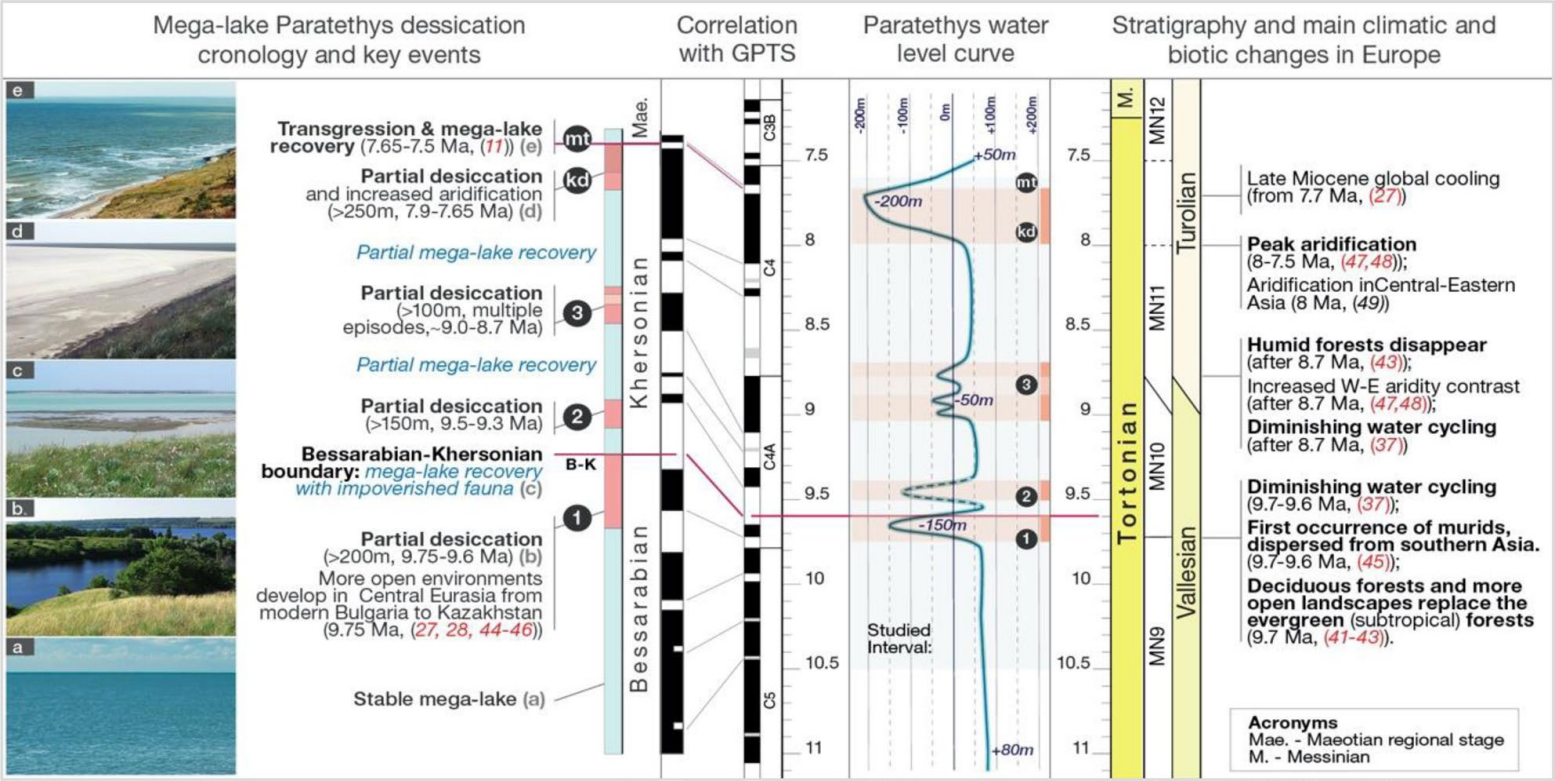
Chronology of the megalake Paratethys hydrological crises, correlations with the Geomagnetic Polarity Time Scale (GPTS 2020^25^), Paratethys water-level curve compared with present-day sea-level in Black Sea (adapted after Popov et al.^7^), stratigraphic correlations (Stratigraphic Stages, Mammal MN zones, European Mammals Zones) and bio-climatic events from neighbouring Europe and Asia. Images on the left exemplify the dynamics of the north Paratethys region: (**a**) submerged lake domain; (**b**) lakes and widespread semi-open landscapes; (**c**) submerged lake domain with some semi-open landscapes; (**d**) dry-arid plains and lagoons; e. submerged lake domain and semi-open landscapes (Image source: Wikimedia commons).

Paleomagnetic measurements allow development of a polarity pattern that can be used to date the partial Paratethys desiccations. The Panagia polarity pattern (Fig. 2, Fig. S6) consists of 17 polarity intervals, 9 of normal polarity and 8 of reversed polarity, plus two additional short polarity fluctuations^23^, that can be correlated with the geomagnetic polarity time scale (GPTS)^24^ and are inferred to correspond to the 11–7.5 Ma interval (Fig. 3, Fig. S6 for a detailed correlation). This correlation provides the first direct age constraints for regression episodes in the Paratethys realm and permits chronological adjustment of the late Miocene Paratethys water level curve of Popov et al.^7^ (Fig. 3).

The first regression episode occurred between 9.75 and 9.6 Ma with a climax at 9.66 Ma (event 1, in Fig. 3, Fig. S6). The next partial desiccation episodes (events 2 and 3, in Fig. 3, Fig. S6) date between 9.5 and 9.3 Ma and between ~ 9.0 and ~ 8.7 Ma. The fourth regression episode began at ~ 8.3 Ma but became more severe between ~ 7.9 and 7.65 Ma. This partial desiccation episode was terminated by the Maeotian transgression (mt) at 7.65 Ma^15,26^, and was linked to a late Miocene global cooling episode^27^.

### Paleogeographic simulations of lake regressions

The late Miocene paleogeographic configuration of the Paratethys realm is poorly known for the 11–7.5 Ma interval. We combine existing paleogeographic reconstructions, lithofacies maps and shoreline data to develop a digital elevation model and simulate water level fluctuations, revealing configurations during the most extreme lake regressions. According to these quantitative paleogeographic reconstructions, we show that the Paratethys had a surface area of more than 2.8 million km^2^ (slightly larger than the present-day Mediterranean Sea) and contained a water volume of more than 1.77 million km^3^ (representing more than 1/3 the volume of the modern Mediterranean), which is larger than any other megalake in the geological record^28^.

Next, we use our 3D paleogeographic model to simulate the maximum regression (~ 280 m water level drop^7^), and resulting paleogeography of the partially desiccated megalake (Fig. 1). In this scenario, the shore significantly retreats on the relatively flat eastern (400–600 km) and northern (100–350 km) margins, while coastlines moderately shift in the western margin (~ 100 km) and the limited steep southern margin (Fig. 1). In addition, regressions fragment the Paratethys into a system of subbasins separated by land bridges. The final paleogeographic configuration implies salt re-distribution in various subbasins, similar to the situation documented during desiccation of Lake Aral^29^. Peripheral basins receive most of the freshwater from precipitation, and outflow from these basins transfers salt to the central basin, a natural process of desalination (Fig. 1). In the heart of the system, deprived of direct discharge from major rivers and fed by salty outflow rivers from peripheral basins, the Black Sea Basin becomes a salt lake, concentrating most of the Paratethys salts^30^. During arid episodes, this central basin will be the most affected, shrinking further as diminished outflow from surrounding peripheral lakes will not compensate for evaporation.

Simulation of the maximum water level drop (to − 200 m) indicates that the remaining lakes would be reduced to 31% of the initial surface and would retain 66% of their initial water volume (Table S2). Simulation of complete desalination of peripheral basins indicates that the Black Sea basin salinity would increase from 12–14‰ in the Paratethys high-stand^16^ to 28.2–32.8‰, which is insufficient to precipitate halite.

### Landscape and environmental changes in inner Eurasia

**Figure 4:**
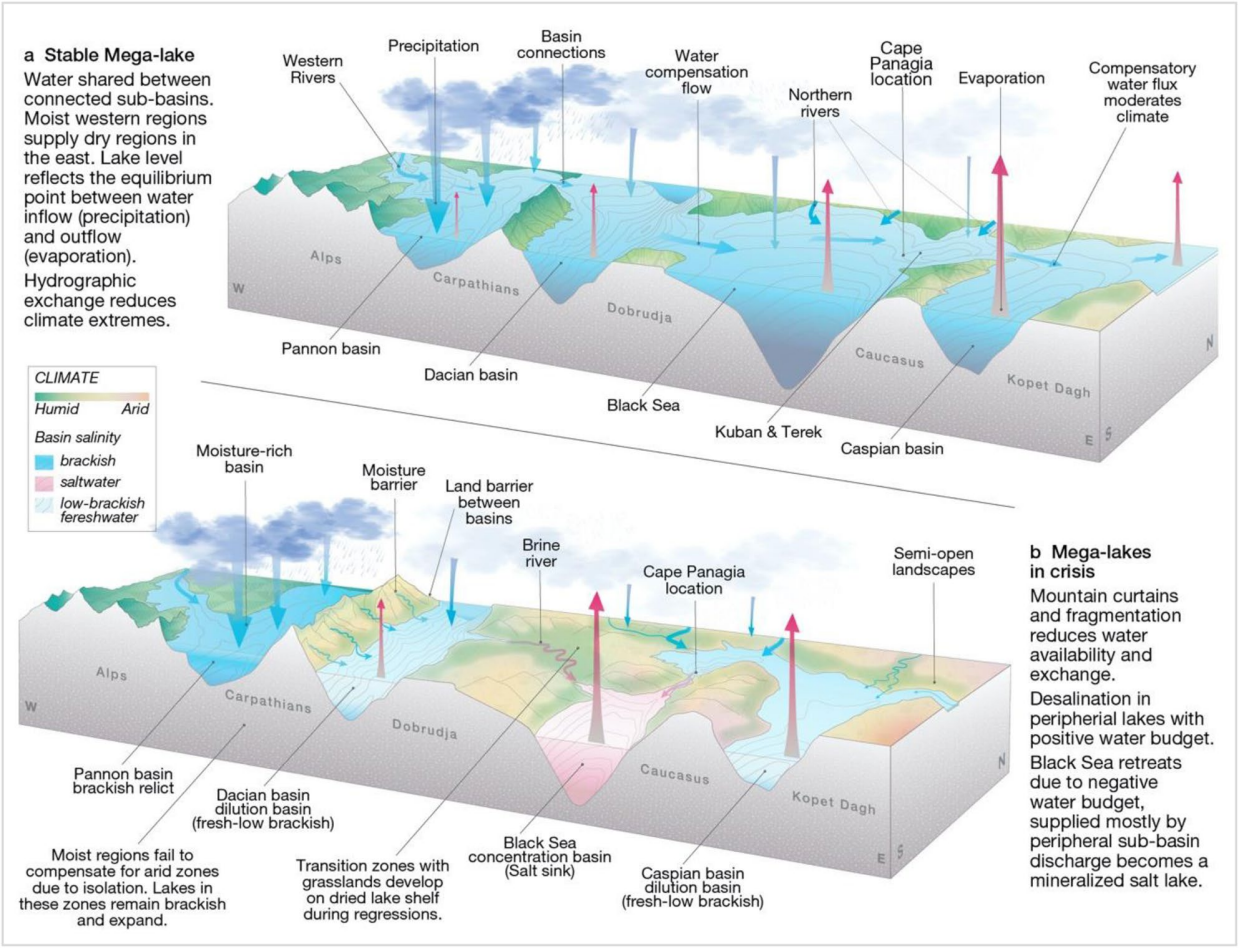
Climate and connectivity impacts on the Paratethys water-basins, a theoretical model illustrating the humid and dry climate impacts on an endorheic Paratethys system. (**a**) Stable system; (**b**) System during partial desiccation episodes.

The Paratethys consisted of a chain of deep sub-basins, connected by shallow zones, distributed roughly along the same latitude (Fig. 1). Due to its isolation from the ocean, the megalake expanded or contracted, determined by the balance between precipitation, river run-off and evaporation. The landlocked paleogeographic configuration made the lake levels prone to water-level instability episodes that occurred when dry or rain belts moved over this latitudinal zone. Initially, water-levels were relatively stable due to the humid regions lying closer to the Atlantic in the west, with positive water budgets that compensated the drier basins of the Eurasian interior (Fig. 4). Disconnection of Lake Pannon in the western periphery led to increased sensitivity to droughts for the remaining Paratethys.

**Figure 5:**
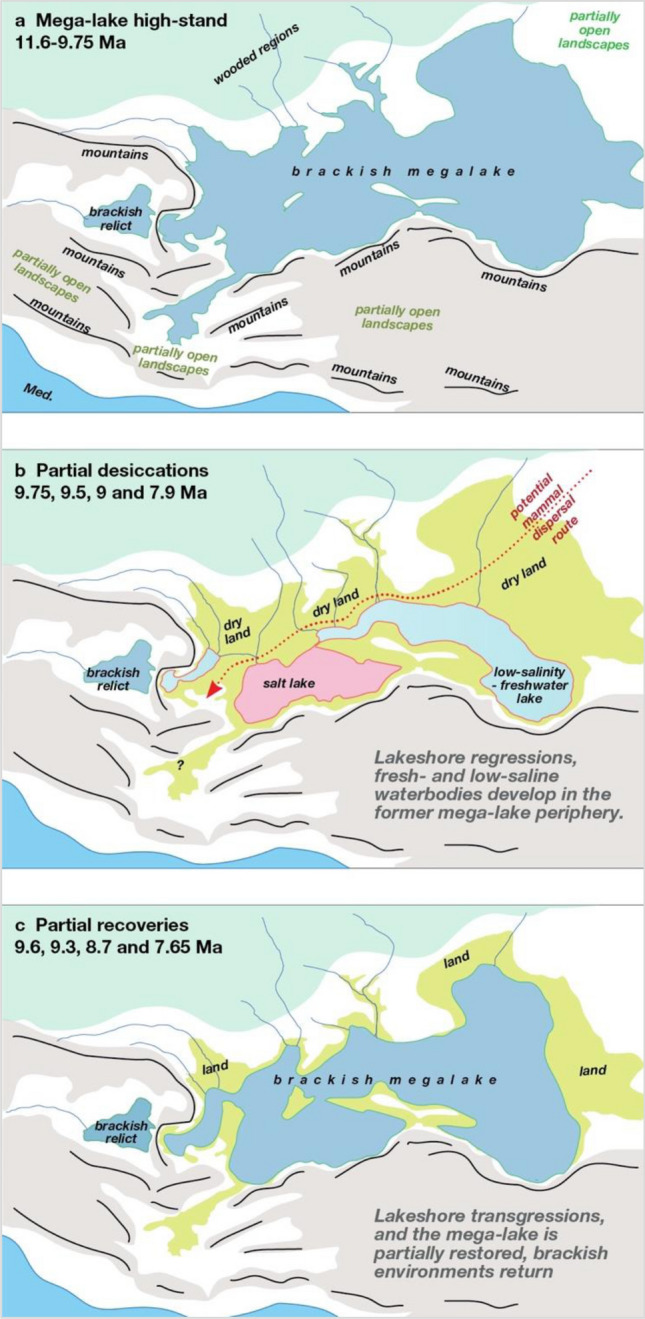
Schematic paleogeographic maps of lake regressions and landscapes of inner Eurasia. Dark green shading represents closed (forest) landscapes, light green shading shows former lake regions transformed to semi-open landscapes and brown shading indicate mountainous regions. Red dotted line indicates potential mammal dispersal routes across dry land and landbridges during lake-level regressions. (**a**) Paratethys megalake during high-stand configuration, (**b**) water distribution during the maximum lake regression. Note that a significant stretch of semi-open landscapes formed in the northern peripheral regions of the former megalake, providing potential passageways for mammal dispersals. (**c**) Paratethys during the partial water level recovery episodes, pushing back open landscapes.

During regression, much of the Paratethys megalake desiccated while remaining sub-basins became independent freshwater, brackish or saline lakes. In a paradoxical twist, regression allowed formation of large desalinated lakes in the Paratethys periphery. A significant area of the Paratethys periphery, particularly the gentle landscape of the northern shelf zone (> 1.75 million km^2^), emerged during regressions and was flooded during transgressions. This ongoing instability hampered permanent development of woodlands, favouring the more flexible grassland-shrub vegetation and giving rise to a late Miocene forest-steppe belt that stretched over 3000 km along the 45° latitude between Central Europe and Central Asia. Pollen records from Panagia indicate that these late Miocene lake regressions corresponded to the expansion of more open landscapes^31^ and increased charcoal in sediments hints at the augmentation of natural fires^32^. Similar late Miocene vegetation changes toward more open landscapes are documented throughout inner Eurasia in Kazakhstan, Ukraine, Moldova^33^ and Bulgaria^34^. Opening of the forest-steppe belt and formation of land bridges during lake regressions (Fig. 5) probably provided an ideal moment of dispersal for mammal populations, living in open Central Asian landscapes through these new passageways to Europe, which in turn would have impacted the food-webs of western Eurasia. Estimations of such dispersals remain poorly constrained because our new age model prompts chronological revision for mammal sites around the Paratethys.

### Lake regressions correlate with environmental changes in Europe and Central Asia

The Paratethys water crises correlate with climate and landscape changes on the continent. At the beginning of the late Miocene, most of inner Eurasia was covered by forests, gradually fading from tropical mixed forests in the south-west to cold conifer forests in the mountains and the far north^35^, except for the East Mediterranean region where drier climates favoured diverse and more open landscapes^36^. Stepwise climatic changes throughout the late Miocene led to increased aridification and seasonality, accompanied by expanding open landscapes (Fig. 3).

The first change occurred at ~ 9.7 Ma, contemporaneous with the onset of Paratethys instability (events 1 and 2 in Fig. 3). An episode of diminished water cycling is observed in Europe in the 9.7–9.5 Ma time interval^37^. In the northern Black Sea region, relatively closed forest environments were replaced by more open environments^38^ and in Panagia, the first evidence of xeric vegetation is documented^39,40^. In western Europe marked decrease of the fruit‐rich evergreen (subtropical) forests and their replacement by deciduous forests and open landscapes began at 9.7 Ma^41–43^, as a consequence of increased seasonality^44^. Fauna changed too: most humidity-adapted and forest-dwelling faunal groups disappeared and immigrants from the peri-Mediterranean and Asia (e.g., murids) arrived in Europe^45^.

The next important climate and paleoenvironmental change occurred at ~ 8.7 Ma, contemporaneous with Paratethys instability (event 3 in Fig. 3). European humid forest landscapes were lost^43^ and replaced by dry, open woodland and grasslands^46,47^, followed by diminished water cycling^37^ and increased aridity^47,48^.

Peak aridity in Europe is documented between 8 and 7.5 Ma^37,47^, while central-eastern Asia experienced increased aridity at ~ 8 Ma^49^, corresponding to the largest lake regression documented in Paratethys (event kd in Fig. 3).Our results fill a blank spot in the chronology of inner Eurasia and Eurasian climate and landscape dynamics. Drought episodes in Europe and Asia correlate with water availability crises in the Paratethys megalake. Paleogeographic simulations provide a basis for future studies that are required to understand the triggers and dynamics of the late Miocene drying of Europe and the role that the inner Eurasian megalake Paratethys played in these events.

## Conclusions

We constrain the age of the late Miocene hydrological crises in the Eurasian megalake Paratethys by integrating sedimentological analyses and magnetostratigraphic dating and resolve four main hydrological crises during the period from 11 to 7.5 million years ago. Using a 3D paleogeographic model, we calculate that the megalake lost ~ 70% of its surface and 1/3 of its volume during regressions, which impacted the climate, hydrology and vegetation of surrounding regions. The model predicts that the remaining water would be split between a central salt lake and peripheral low-salinity or freshwater lakes. Emergent land was preconditioned to become forest-steppe belts that better connected Central Asia with Europe and allowed terrestrial faunal exchanges between east and west. In an endorheic state, the Eurasian Paratethys megalake became unstable and experienced partial desiccation episodes. These events correlate stepwise drying and landscape opening in Europe with dry climate episodes in central Asia, which raises questions about whether the Paratethys crises were trigger, contributor or mere expressions of dry climatic episodes in Europe and central Asia.

## Materials and methods

### Sedimentology

We focus here on a ~ 500 m thick stratigraphic interval containing the Khersonian regional sub-stage^16^ that was logged, sampled and mapped in high-resolution. Detailed sedimentological studies focused on the uppermost Bessarabian–Khersonian–Maeotian interval of Panagia (Fig. S4) and are based on field observations focused on lithofacies descriptions (lithology, grain-size, colour, sedimentary structures) and associated trace fossils. The descriptive terminology follows the field guide of Tucker^50^, while the general facies concept is based on Miall^51,52^. Later, commonly occurring lithofacies were combined into eight distinct facies associations following the concept of Collinson^53,54^. Facies associations were correlated to specific depositional environments based on sedimentological reasoning and comparison with literature examples (Table S1).

### Paleomagnetism

Magnetostratigraphy can provide age dating for rock successions if the established polarity pattern of studied sections can be correlated to the reversal pattern of the Geomagnetic Polarity Time Scale^24^ (GPTS). This approach has proven successful with Paratethys sediments, when adequate demagnetization techniques are applied to deal with the generally high concentration of iron sulphides in anoxic sediments, and particularly with the magnetic mineral greigite^55,56^.

The Panagia section is a cliff affected by active coastal erosion and erosion-induced instability. We drilled and oriented in situ 694 paleomagnetic samples, corresponding to 347 discrete stratigraphic levels (Fig. 2), and measured bedding planes for each sample level.

Samples corresponding to distinct lithological units have been subjected to thermomagnetic measurements to determine the chemical nature of the magnetic carrier and to identify the most suitable heating profile for thermal demagnetization.

#### Rock magnetism

Thermomagnetic measurements of the induced magnetization (J-T curves) in air at high temperatures were conducted with a modified horizontal translation-type Curie balance with a sensitivity of ~ 5 × 10^–9^ Am^2^^57^. A field, cycled between 100 and 300 mT, was applied to powdered sediment samples (~ 70 mg). Multiple heating (6°/min) and cooling runs (10°/min) were performed between room temperature and steps of 150 °C, 250 °C, 350 °C, 450 °C, 525 °C and 700 °C. In terms of rock-magnetic properties (Fig. 2b), we divided the samples into three types: type 1 is characterized by the presence of greigite, indicated by the typical irreversible magnetization decrease after heating to ~ 320 °C and pyrite as indicated by a magnetization increase after heating to 380–420 °C (due to its oxidation)^58^. Type one samples are rare (less than 5%). Type 2 samples are characterized by weak iron oxides. The presence of pyrite indicates that reductive dissolution of iron oxides has occurred, which causes lower magnetisations of sediments^59^. These samples are abundant and are mostly found in the Bessarabian and lower Khersonian, suggesting a stratified environment and frequent bottom water anoxia. Finally, type 3 samples are characterized by iron oxide magnetic carriers. Sulfides such a pyrite and greigite are lacking in these samples found in carbonatic beds and the upper Khersonian continental levels.

#### Magnetostratigraphy

We use progressive thermal demagnetization and/or progressive alternating field demagnetization to isolate the characteristic remanent magnetization (ChRM). The natural remanent magnetization (NRM) was thermally demagnetized and measured using a 2G Enterprises DC Superconducting QUantum Interference Device (SQUID) cryogenic magnetometer (noise level of 3 × 10^−^^12^ Am^2^). Heating was performed in a laboratory-built, magnetically shielded furnace, with a residual field less than 10 nT. The presence of pyrite, indicated by J-T runs, informed the thermal demagnetization runs strategy, which were limited to a maximum temperature level of 380 °C. Due to pyrite oxidation at 420 °C, we restricted heating to well below this point (380 °C) to avoid pyrite oxidation artefacts from contaminating our results. Also, alternating field demagnetization was performed, with small field increments, up to a maximum of 100 mT with an in-house robotized sample handler, attached to a horizontal 2G Enterprises DC SQUID cryogenic magnetometer^60^.

Thermal and alternating field demagnetization of Cape Panagia samples reveal a weak, low-temperature (LT), viscous overprint that is generally removed at 150 °C and 15 mT (Fig. 2a). A second higher temperature (HT) component that we consider to correspond to the ChRM is demagnetized at temperatures between 120 and 380 °C or fields between 15–45 mT. This component is averaged from four or more consecutive temperature/field steps and calculated using principal component analysis^61^. We obtained ChRM directions for 614 samples of both reversed and normal polarities.

We used only one sample per level for plots and statistics, discarding duplicates with lower mean angular deviation (MAD). The 456 samples that resulted after the selection are assigned to four qualitative groups. The highest quality directions represent samples with MAD values (unanchored) below 6° and are attributed to group Q1 (Fig. 2, black dots—164 specimens). Average quality directions represent group Q2 (Fig. 2, purple diamonds—167 specimens) and comprise samples with abnormal orientations and MAD values between 6° and 12°. The third group Q3 represents poor quality directions with MAD > 12° (Fig. 2, labelled with lilac diamonds—62 specimens). A fourth group X (Fig. 2, labelled with grey x-es—63 specimens) contains specimens that do not show consistent directions. Only Group Q1 and Q2 samples have been used to develop a polarity pattern (totalling 331 specimens) for the Cape Panagia section. The remaining samples from groups Q3 and X (totalling 125 specimens) have been discarded from polarity interpretation but are nevertheless plotted to give a full picture of ChRM estimates for the Cape Panagia record. Group X samples are plotted as zero values for the sole purpose of highlighting areas with problematic ChRM preservation.

The final polarity pattern of the Cape Panagia section consists of 17 intervals, 9 of normal polarity, and 8 of reversed polarity (Fig. 2, Fig. S6) and two thin polarity intervals (1 × and N1y, of reversed polarity (in the N1 zone) that could represent short-term polarity fluctuations^23^. The lowermost part of the section (120–250 m) comprises a long normal polarity interval (N1) that we tentatively correlate to the long normal polarity chron C5n.2n of the GPTS (Fig. S6). The short events N1x and N1y could then correspond to short-term polarity fluctuations C5n.2n-1 and C5n.2n-2^62^. Upward tuning subsequently correlates N2 to C5n.1n and N3 to C4Ar.2n, which places the Bessarabian-Khersonian boundary (310 m) at 9.6 Ma. The long dominantly reversed polarity interval between 360 and 420 m most likely correlates to C4Ar. We detected two short normal polarity zones: N4, which correlates to C4Ar.1n (N4) and N5, which correlates to the short-term polarity fluctuation C4Ar.1r-1^23^. Next, we associate normal polarity interval N6 with C4An, N7 with C4r.1n, and the long normal polarity interval N8 with C4n.2n (Fig. S6). This correlation implies that the Khersonian–Maeotian boundary is located in chron C4n.1r at 7.65 Ma, in agreement with previous studies^15,16,26^.

Sediment accumulation rates for the Cape Panagia section (Fig. S6) have a marked change in the middle Khersonian. Relatively high average sedimentation rates (~ 25 cm/kyr) characterize the older deposits that precede the water-level instability phase (10.4–9.1 Ma). Lower sedimentation rates (~ 10 cm/kyr) are typical in the upper interval (9.1–7.65 Ma). In both cases, sedimentation rate increases are associated with hydrological crises identified from biostratigraphic and sedimentological observations.

Magnetostratigraphic correlation allows us to develop an age model for late Miocene hydrological instability events identified by paleontological, lithological and sedimentological investigations. The terminal Bessarabian regression (event 1 in Fig. 2, Fig. S6) corresponds to the time interval between 9.75 and 9.6 Ma, with a climax at 9.66 Ma. The next two instability episodes (2 and 3 in Fig. 2, Fig. S6) date between 9.5 and 9.3 Ma and between ~ 9.0 and ~ 8.7 Ma. The Paratethys megalake experienced a maximum regression during the Great Khersonian Drying (kd in Fig. 2, Fig. S6) that occurred between ~ 8.3 and 7.65 Ma, followed by the Maeotian transgression (mt) at 7.65 Ma.

For the Panagia section, the reversal and Watson tests^63,64^ give negative results, due to high ChRM directions scatter which makes the angle γ between mean normal and reversed polarities larger than the critical angle γ_c_. Paleomagnetic directions (declinations) for the normal polarity samples from Group Q1 have an average declination of ~ 346°, suggesting a counter-clockwise rotation of 14°, which is significantly different from the 14° clockwise rotation revealed by the average declination of reverse polarity samples (~ 194°). The one explanation for this result is the incomplete removal of a secondary NRM component as observed by other authors on similar sections where multiple magnetic carriers are present^65–67^. Overlapping unblocking spectra may also lead to overestimation of inclinations for normal polarity directions and, hence, to a negative reversal test. However, this does not disqualify the magnetostratigraphic results. The polarity results from Panagia are robust with clustered ChRM directions that define a complex pattern that can be correlated with the GPTS and that is in agreement with patterns documented at other sites throughout Paratethys for the end Khersonian-Maeotian interval^15,26,66,68^.

Inclination values (36.7°) are lower than expected for the late Miocene position of Taman^69^, which suggests that the Cape Panagia sediments have been subjected to inclination shallowing processes like compaction and de-watering (Fig. S7). We use the E-I method^70^ on the online platform Paleomagnetism.org^71^ to detect and correct for inclination shallowing of the Panagia sample directions (after tectonic correction). The average inclination changes from 36.7° (before unflattening) to 61.9° (after unflattening) (Fig. S7).

### Paleogeographic reconstructions

Paratethys paleogeography has been the subject of intense research in recent decades resulting in local paleogeographic maps^9,72–74^, tectonic reconstructions^75^ and regional paleogeographic maps^4,15^. Popov et al.^6,8,20,76^ recompiled and refined local maps in a series of paleogeographic and paleo-facies maps for the Paratethys region, supplemented by shoreline reconstructions for platform regions of southern Russia and Ukraine^7^. However, these reconstructions generally avoid the time interval between 11.6 and 7.2 Ma.

To reconstruct the paleogeography in this interval, we present two digital elevation models (DEM), based on the late Serravallian (> 11.6 Ma—Map 6) and late Tortonian-early Messinian (~ 7.2 Ma Map 7) reconstructions of Popov. The first DEM is used to estimate the maximum megalake expansion (Fig. 5a). The second DEM (Fig. 5c), closer to the largest partial megalake desiccation episodes (~ 8–7.65 Ma), is used to simulate the water-level drop and obtain the partially desiccated megalake paleogeography (Fig. 5b).

These Paratethys paleogeographic reconstructions are complemented by paleogeographic data from a range of regional studies of northern Europe^77^, western Europe^78^, Alps and Central Europe^79^, Pannonian basin^80^, Gibraltar region^1,2^, Aegean Sea^4^, Marmara Sea paleobathymetry^81,82^, Mediterranean paleobathymetry and paleotopography^5^ and a Middle East tectonic map^83^.

Reconstructing the paleogeography of the Paratethys realm required conversion from facies on existing paleogeographic maps to bathymetry, based on digitizing facies zones and converting them to depths using facies-depth estimations and references from the modern analog Black Sea, Azov Sea, and Caspian Lake. For the Black Sea and Caspian Lake basins, maximal depth zones are based on analogies with present-day bathymetry of the above-mentioned basins.

To generate a Paratethys DEM, we used the ANUDEM method applied in ESRI ArcGIS Pro, version 2.1^84^, which calculates topographical surfaces sensitive to shape and drainage structure resulting in hydrologically correct models. The model was then used to calculate the total water volume. Subsequently, DEMs for various base-level drops were obtained by applying simple map algebra additions and subtractions to the Parathetys DEM. For each newly created DEM, the volume, 2D area and 3D area were estimated below specific thresholds. This was accomplished using the Surface Volume tool from ArcGIS Pro, version 2.1^85^.

## Data availability

All data are available in the main text or supplementary materials.

## Supplementary Information

Supplementary Information 1.

Supplementary Information 2.
