# Supplementary material for: Late Miocene megalake regressions in Eurasia
Source: Sci Rep. 2021 Jun 1;11:11471. doi: 10.1038/s41598-021-91001-z (PMC8169904; doi:10.1038/s41598-021-91001-z)
Supplement: Supplementary file 1 — Supplementary Information 1. [file 41598_2021_91001_MOESM1_ESM.docx]

**Supplementary Information for**

**Late Miocene megalake regressions in Central Eurasia**

Dan V. Palcu^a,b,**^, Irina S. Patina ^c ,*^, Ionuț Șandric^d^, Sergei Lazarev^a^, Iuliana Vasiliev^e^, Marius Stoica^f^, Wout Krijgsman^a^

Irina Patina

[irina.patina@gmail.com](mailto:irina.patina@gmail.com),

Dan V. Palcu

[d.v.palcu@uu.nl](mailto:d.v.palcu@uu.nl)

**This PDF file includes:**

Figures S1 to S7

Tables S1 to S2

Legends for Datasets S1

**Other supplementary materials for this manuscript include the following:**

Datasets S1


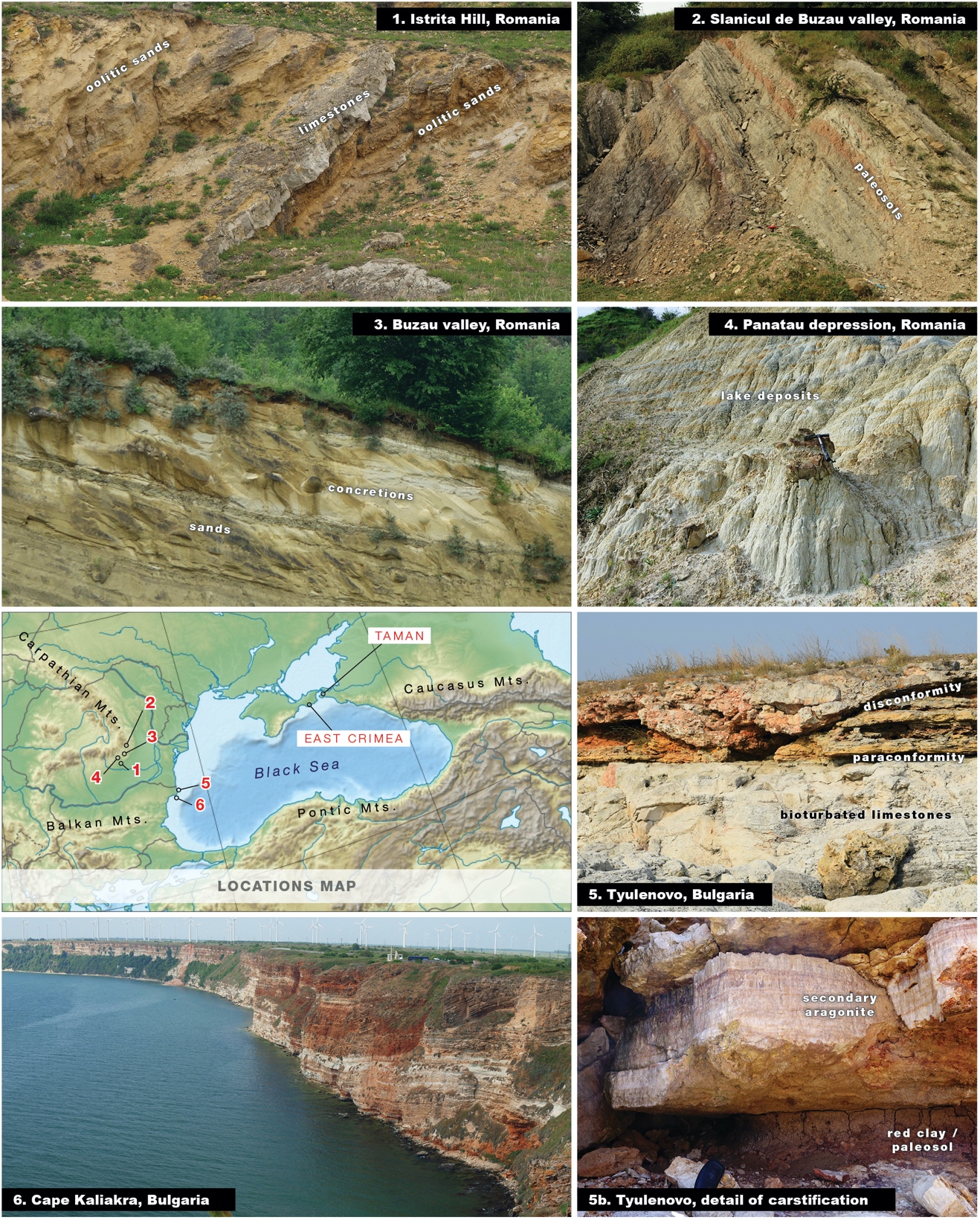
Fig S1. Sedimentary records that proved to be unsuitable for developing an age model for late Miocene hydrological fluctuations of Paratethys. 1. Istrița, Romania, carbonate platform, coastal and continental deposits; 2. Slănicul de Buzău, Romania, coastal-continental deposits; 3. Buzău valley, Romania, massive sand deposits; 4. Subcarpathians, Romania, freshwater lake deposits; 5. Tyulenovo, Bulgaria, carbonate platform with erosion and karstification levels - detail is of paleocarst fill deposits and secondary aragonitic deposits; 6. Cape Kaliakra, Bulgaria, carbonate platform with erosive and karstification levels. (Photos: D.V. Palcu, Map: Europe laea topography, Wikimedia commons).


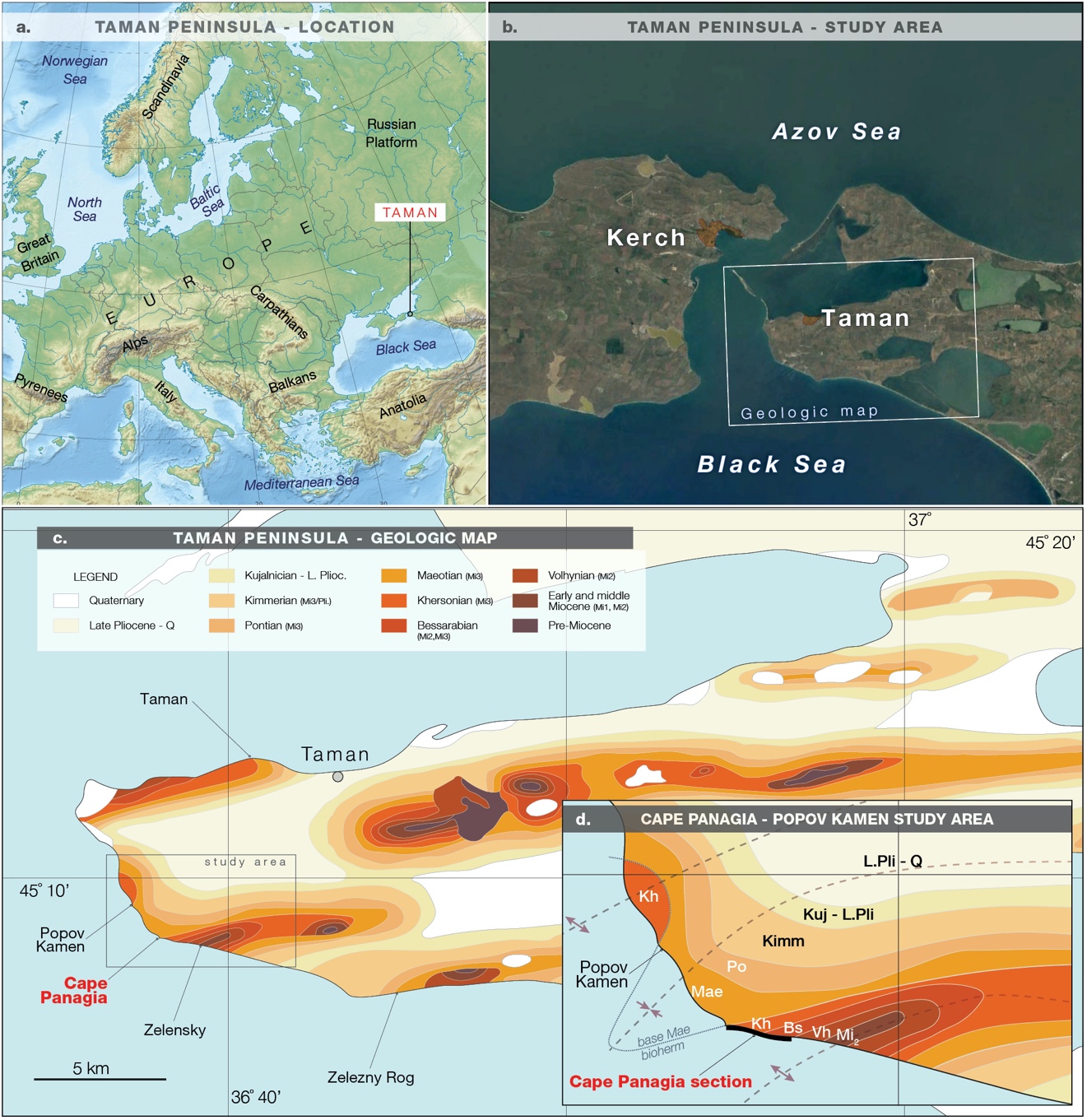
Fig. S2. Geographic and geologic context of the Cape Panagia section. a. Location of the Taman Peninsula (Map: Europe laea topography, Wikimedia commons); b. location of the area of interest in the Taman Peninsula (map: Google Earth); c. Geological map of the area of interest in the Taman Peninsula; d. detail of the geological context of the Panagia section (map: Geological map of the Russian Federation, 2001^1^).


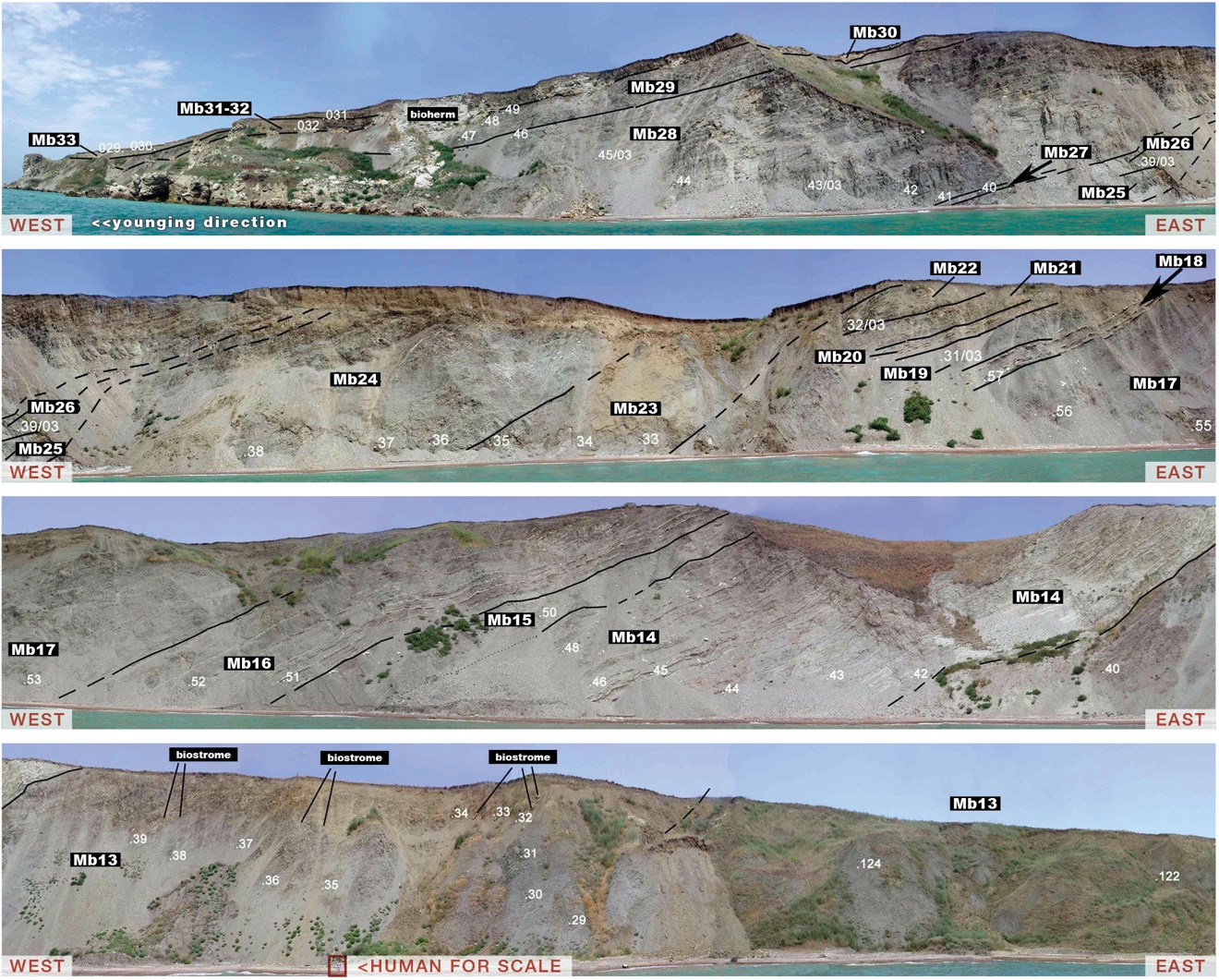
Fig. S3. Overview of the Cape Panagia section (photos courtesy of Sergey V. Popov). The “Mb” annotations stand for the lithological sub-units, while numbers represent sampling points (both sensu- Popov et al.(2017)). Please note the human for scale, in the lowermost image.


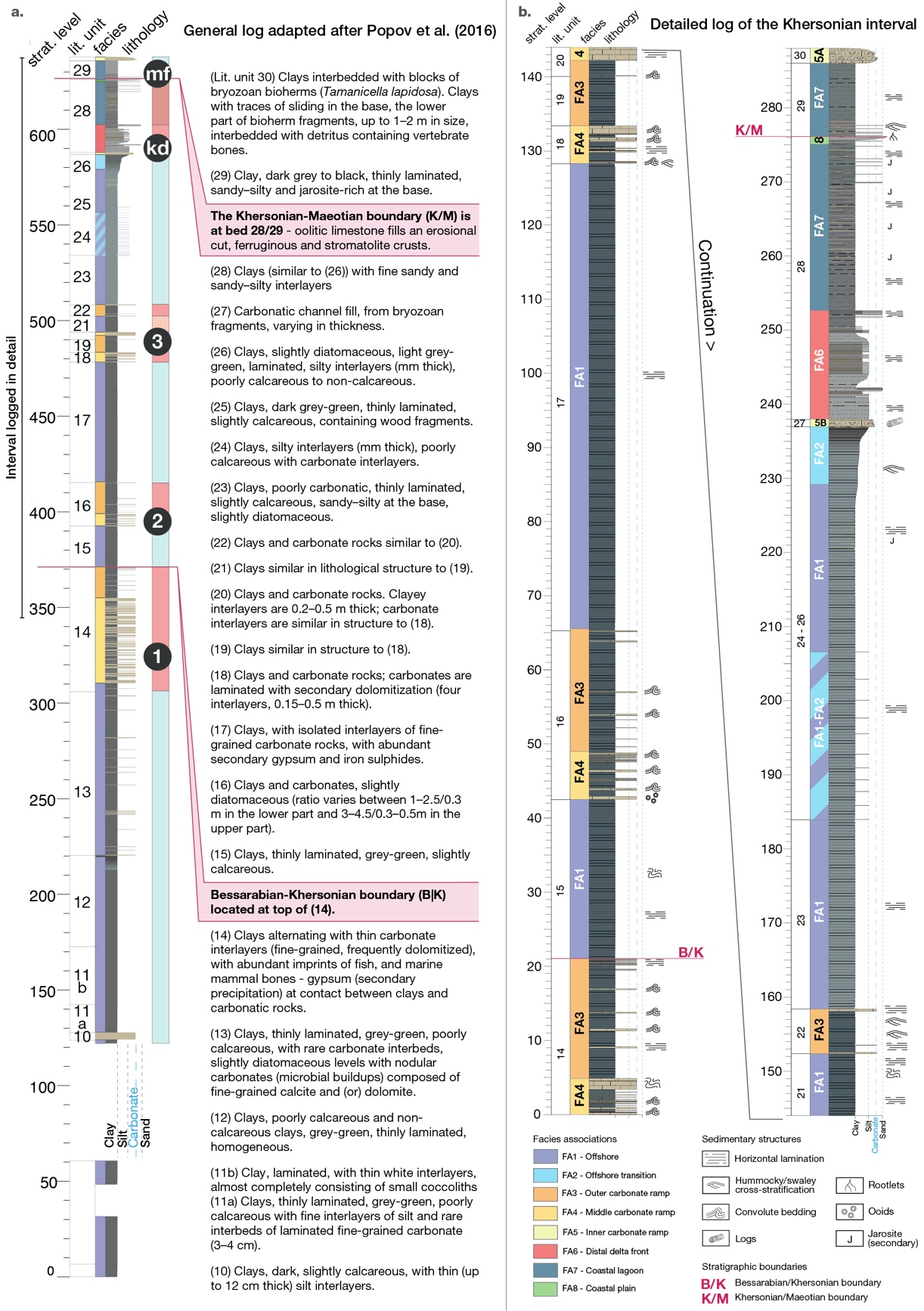
Fig. S4. Lithological and paleontological characterization of the Cape Panagia sedimentary succession. a. Original data (after Popov et al. (2017)), b. Sedimentological observations presented here.


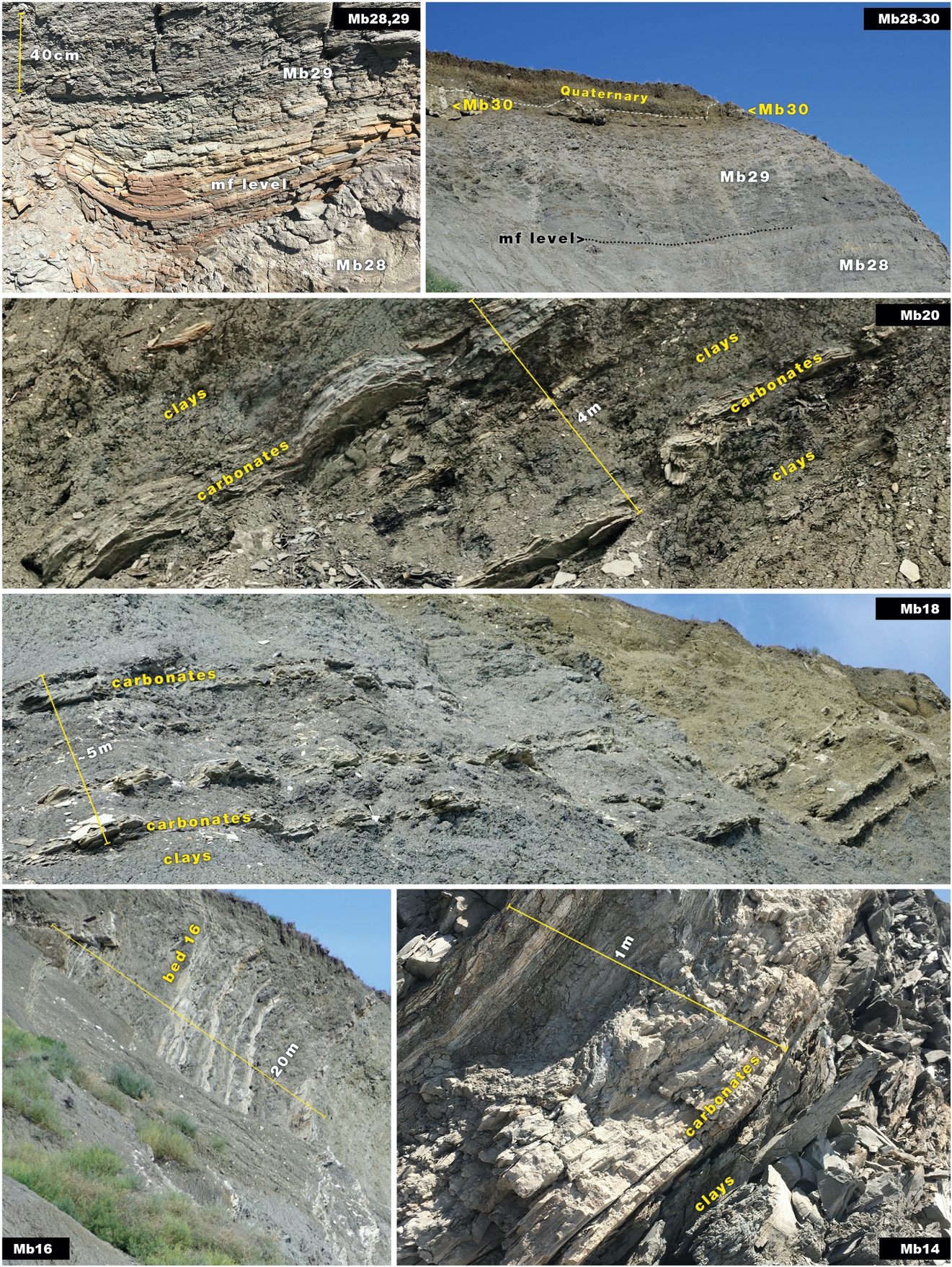
Fig S5 – Carbonate-rich sediments and the Maeotian flood level (mf) in the Panagia section. (Photos: D.V. Palcu)


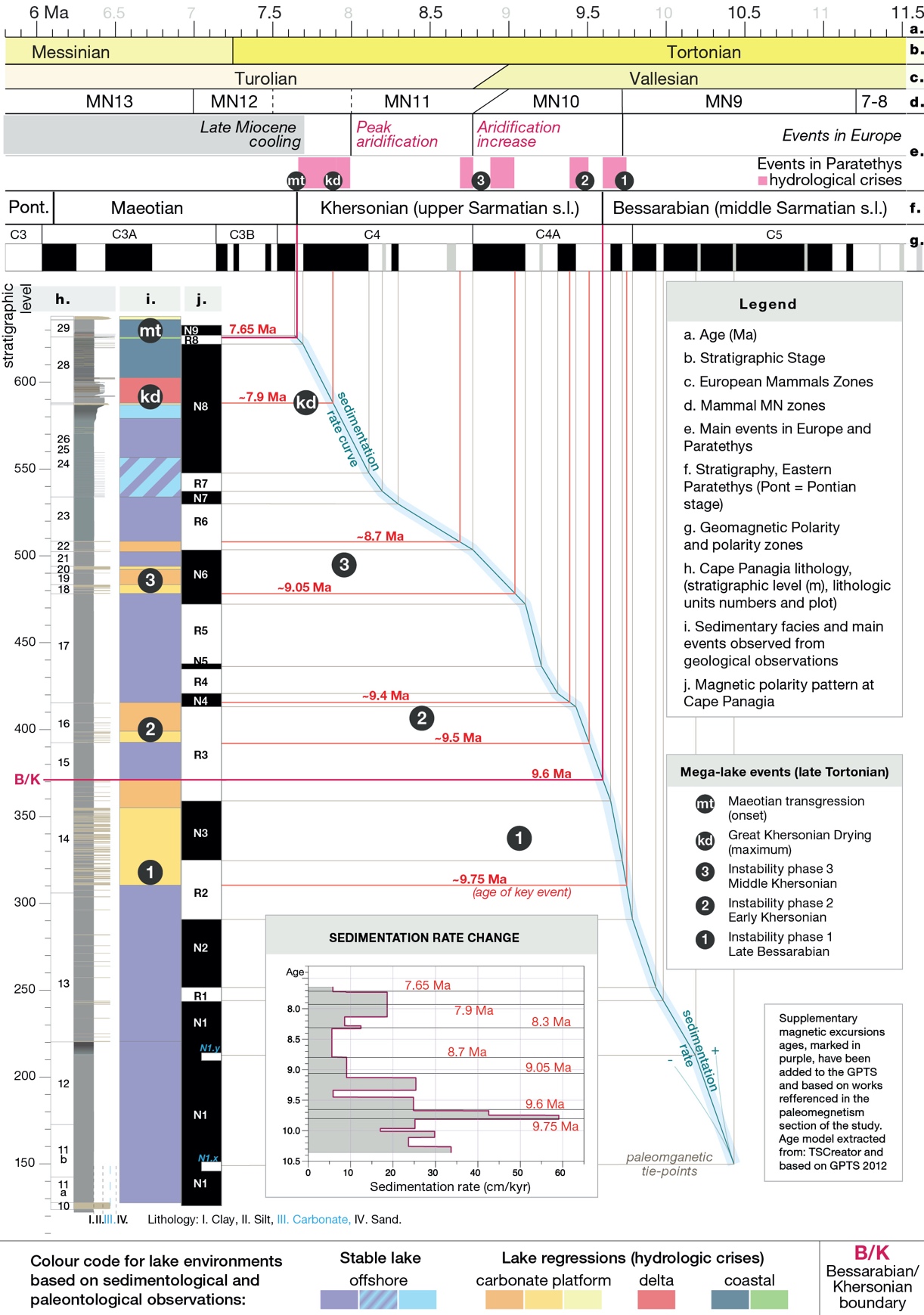
Fig. S6. Geochronology of Paratethys hydrological crises. Synthetic site descriptions and correlations to the Geomagnetic Polarity Time Scale (GPTS 2012) and to the main climate changes in Europe.


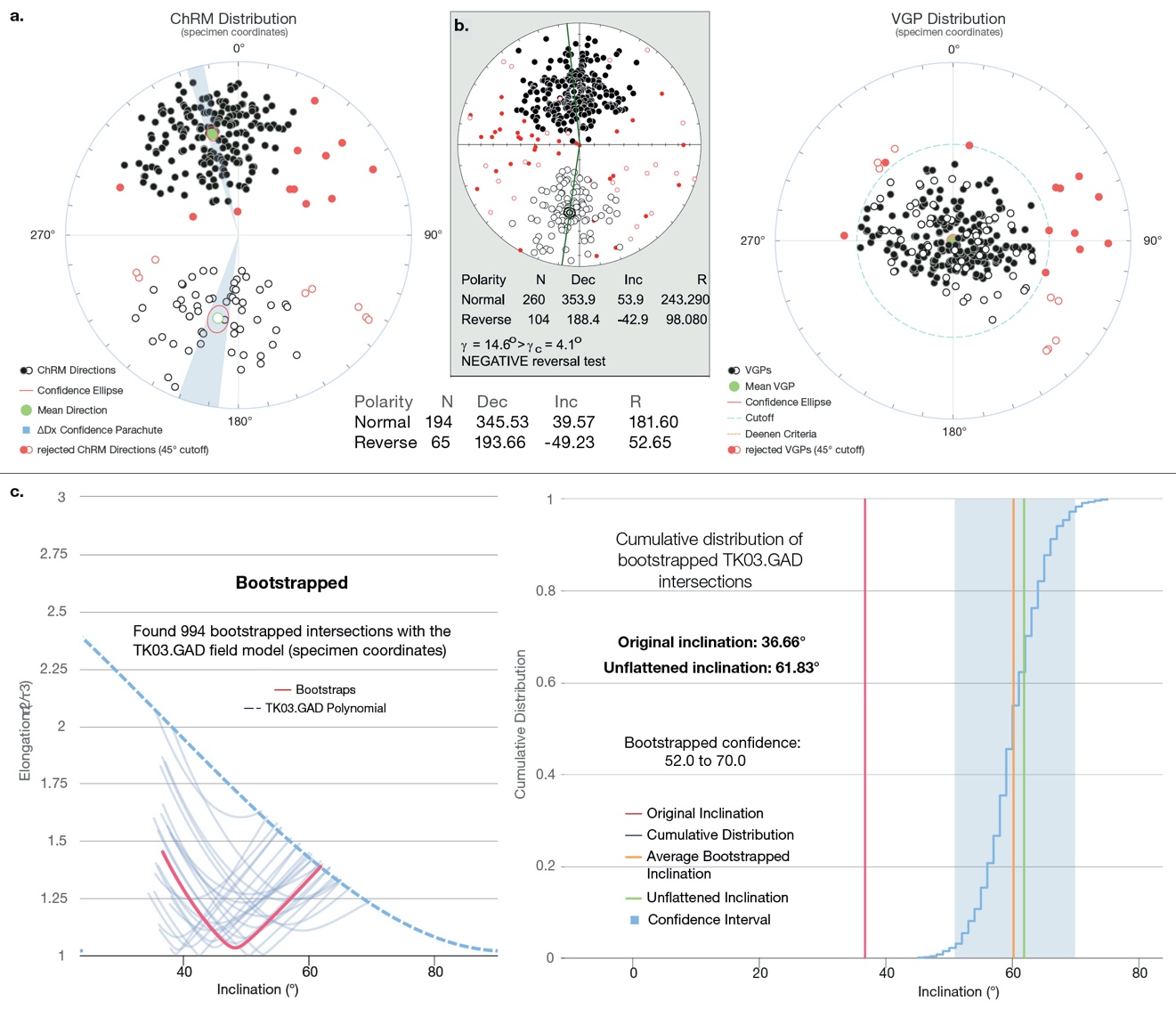
 Fig. S7. Supplemental paleomagnetic data. a. Equal area projection of ChRM directions in tilt-corrected coordinates for the samples giving the straightforward directions in Fig. 2. Solid (open) circles indicate normal (reversed) polarity and are the projections on the lower (upper) hemisphere. Red (small) circles indicate the individual directions rejected by the 45° cut-off angle; b. Similar results were obtained from the nearby Zelezny Rog section^2^ (Fig S2); c. inclination unflattening calculations.

| FA1: Grey, dark grey slightly calcareous claystones, thinly (1-4 mm) parallel laminated. FA1 usually passes into FA2 and FA3 with a gradual contact. | Offshore. Accumulation from suspension fallout in a stable water column, below storm wave-base ^2^. Offshore depositional environments are ubiquitous in littoral successions and were reported from the Upper Cretaceous of the Back Tongue^3^. |
| --- | --- |
| FA2: Grey, greenish-grey parallel-laminated to hummocky cross-stratified slightly calcareous claystone with thin (1-5 mm) siltstone layers. FA2 passes into FA1 with gradual contact or FA5 with a sharp erosional contact. | **Offshore transition.**Deposition above storm-wave base as indicated by hummocky cross-stratification^4,5^. |
| FA3: Grey parallel-laminated, locally hummocky cross-stratified calcareous claystones with thin (up to 4 cm) layers of pale yellow, white, fine-grained limestone and wackestone. Carbonate layers frequently above convolute structures. FA3 gradually develops either into FA4 or into FA1. | **Outer carbonate ramp**. Thin laminae and fine-grained carbonate layers in claystone-dominated packages reflect accumulation in distal outer carbonate ramp environments^6^. Locally developed hummocky-cross stratification suggests that the outer ramp occasionally reached storm wave-base^2,6^. Outer carbonate ramp depositional environments with similar facies are known from Late Kimmeridgian of North-western Spain (e.g., Facies 1^7^). |
| FA4: Pale yellow sharp-based parallel-laminated, convoluted, locally hummocky cross-stratified wackestones and packstones in sharp 0.05 – 1.9 m beds interbedded with dark grey parallel-laminated calcareous claystones in beds up to 1 m. FA4 gradually passes into FA3 or FA1 with a sharp contact. | **Middle carbonate ramp.**Deposition above storm-wave base, based on frequent hummocky-cross stratification and convolute bedding^4,5^. The prevalence of packstones and the presence of ooidic sandstones along with fine-grained limestones (wackestones) point to a mixture of autochthonous and allochthonous carbonates^6^. Similar middle ramp facies are described in the Kimmeridgian carbonate ramp of the Iberian Ranges^8^. |
| FA5A: Horizon with chaotically distributed isolated build-ups (up to 7 m FA 7) of bryozoan bioherms. FA5B: Irregular sharp-based rudstone (up to 2m thick) consisting of 2-5 cm bryozoan and peloid fragments, with rare cobbles and wood fragments. The upper contact of FA5A is not exposed while FA5B is followed by FA6 with a sharp contact. | **Inner ramp**. Shallow water deposition above fair-weather wave-base^6^. Bryozoan bioherms commonly appear during sea-level rise^2^. Well sorted rudstone with erosive bases represent downslope mass-transport from a shallow part of the ramp and could be linked with a regression^6^. Lack of matrix and well sorted rudstone may be indicative of wave reworking. Similar lithofacies are interpreted in Miocene inner ramp deposits of the Central Apennines ^9^. |
| FA6: Thinly-interbedded grey parallel-laminated siltstones in beds 0.03 – 0.3 m, brown parallel-laminated mudstone in beds 0.03 – 0.5 m and thin (up to 5 mm) very fine-grained sandstone layers. FA6 gradually transforms into FA7. | **Distal delta front.**Products of river discharge in the near-cast area behind the inner ramp. Grain size variation depends on river discharge intensity, with sandstone representing heavily sediment-loaded outflow events and siltstone/mudstone reflecting fallout from a lighter suspended fraction^10^. Within carbonate ramps, siliciclastic deltaic packages are described in the Carnian deposits of Lombardy^11^. |
| FA7: Dark grey massive, parallel-laminated, locally wave-ripple cross-laminated claystone, with thin stripes (1-3 mm thick) of very fine-grained sandstones. Locally, with thin (up to 0.05 m) layers of pale yellow, white diatomite (?) and yellow powder of secondary jarosite. FA7 gradually passes into FA8. | **Coastal lagoon.** Accumulation in the transitional zone between the inner ramp and coastal plain. Dominance of organic-rich claystone points to a sheltered lagoon that traps organic material (Allen and Johnson, 2011). Sandstone lenses and stripes, along with wave ripple marks, indicate wave activity. Similar interpretation of siliciclastic lagoonal deposits developed behind the inner ramp are known from the Carnian deposits of Lombardy (Italy)^11^. |
| FA8: Grey, structureless muddy fine-grained sandstone (0.5 mthick) with mm-scale rootlets, disturbed by incised small-scale channels (1 m deep, 0.5 m wide). FA8 is followed by FA7 with a sharp erosional contact. | **Coastal plain.** Gradually developed structureless sandstone with mm-scale rootlets suggests deposition on the daylight surface. The coastal plain is the shallowest member of the carbonate ramp successions and are described in the Carnian deposits of Lombardy (Italy)^11^. |

Table S1. Facies descriptions of stratigraphic intervals corresponding to the hydrological crises, based on field observations.

| Basin | Age  (Ma) | Surface (km^2^) | Volume  (km^3^) | Salinity  (‰) | Salinity  (‰) |
| --- | --- | --- | --- | --- | --- |
| Paratethys – maximum extent  *(before fragmentation, +80 m)* | ~12 | 2,839,545 | 1,774,795 | 12.00* | 18 |
|  |  |  |  |  |  |
| Paratethys mega-lake  *(after fragmentation and loss of the Pannon Lake region, +80 m)* | ~10 | 2,573,633 | 1,679,881 | 12.00* | 14.00* |
|  |  |  |  |  |  |
| Salinity simulations: |  |  |  |  |  |
| Remaining lakes *(partial desiccation of 280 m)* | 7.9 | 807,709 | 1,104,368 | 0 | 0 |
| Black Sea basin *(-200 m)* | 7.9 | 354,898 | 716,177 | 28.15 | 32.84 |

Table S2. Quantitative estimation of Paratethys hydrological crises. Volumes and surfaces of the mega-lake and sub-basins extracted from a 3D paleogeographic model and salinity simulations based on initial estimations of salinities^12^ the assuming of a salt conservation.

Dataset S1 (separate file). Concise paleomagnetic results

Bibliography

1. Korsakov, S. G. & Zarubin, V. V. The 1: 200000 Geological Map of the Russian Federation, Ser. Caucasus, sheets L-37-XIX, L-37-XXV (Taman) [in Russian]. (2001).

2. Reading, H. G. Sedimentary environments: processes, facies and stratigraphy. 688 (1996).

3. Yoshida, S. Sequence and facies architecture of the upper Blackhawk Formation and the lower Castlegate Sandstone (Upper Cretaceous), Book Cliffs, Utah, USA. *Sediment. Geol.* **136**, 239–276 (2000).

4. Cheel, R. Hummocky and swaley cross-stratification. in *Sedimentology, Encyclopedia of Earth Science* 585–588 (Springer Berlin Heidelberg, 1978).

5. De Raaf, J. F. M., Boersma, J. R. & Van Gelder, A. Wave‐generated structures and sequences from a shallow marine succession, Lower Carboniferous, County Cork, Ireland. *Sedimentology* **24**, 451–483 (1977).

6. Flügel, E. Microfacies of carbonate rocks: analysis, interpretation and application. *Choice Rev. Online* **42**, 42-3437-42–3437 (2005).

7. Bádenas, B., Aurell, M., Rodríguez-Tovar, F. J. & Pardo-Igúzguiza, E. Sequence stratigraphy and bedding rhythms of an outer ramp limestone succession (Late Kimmeridgian, Northeast Spain). *Sediment. Geol.* **161**, 153–174 (2003).

8. Bádenas, B. & Aurell, M. Proximal-distal facies relationships and sedimentary processes in a storm dominated carbonate ramp (Kimmeridgian, Northwest of the Iberian Ranges, Spain. *Sediment. Geol.* **139**, 319–340 (2001).

9. Corda, L. & Brandano, M. Aphotic zone carbonate production on a Miocene ramp, Central Apennines, Italy. *Sediment. Geol.* **161**, 55–70 (2003).

10. Fielding, C. R. Planform and facies variability in asymmetric deltas: Facies analysis and depositional architecture of the Turonian Ferron Sandstone in the western Henry Mountains, south-central Utah, U.S.A. *J. Sediment. Res.* **80**, 455–479 (2010).

11. Gnaccolini, M. & Jadoul, F. Carbonate platform, lagoon and delta ‘high-frequency’ cycles from the Carnian of Lombardy (Southern Alps, Italy). *Sediment. Geol.* **67**, 143–159 (1990).

12. Popov, S. V., Antipov, M. P., Zastrozhnov, A. S., Kurina, E. E. & Pinchuk, T. N. Sea-level fluctuations on the northern shelf of the Eastern Paratethys in the Oligocene-Neogene. *Stratigr. Geol. Correl.* **18**, 200–224 (2010).
